# Mechanoregulation and pathology of YAP/TAZ via Hippo and non-Hippo mechanisms

**DOI:** 10.1186/s40169-018-0202-9

**Published:** 2018-08-13

**Authors:** Oleg Dobrokhotov, Mikhail Samsonov, Masahiro Sokabe, Hiroaki Hirata

**Affiliations:** 1 R-Pharm Japan, Tokyo, 105-0001 Japan; 2 Mechanobiology Laboratory, Nagoya University Graduate School of Medicine, 65 Tsurumai, Showa-ku, Nagoya, 466-8550 Japan; 3 R-Pharm, Moscow, 123317 Russia

**Keywords:** YAP, TAZ, Hippo pathway, Mechanotransduction, Contact inhibition of proliferation, Actomyosin, Cancer progression, Cancer therapy resistance

## Abstract

Yes-associated protein (YAP) and its paralog WW domain containing transcription regulator 1 (TAZ) are important regulators of multiple cellular functions such as proliferation, differentiation, and survival. On the tissue level, YAP/TAZ are essential for embryonic development, organ size control and regeneration, while their deregulation leads to carcinogenesis or other diseases. As an underlying principle for YAP/TAZ-mediated regulation of biological functions, a growing body of research reveals that YAP/TAZ play a central role in delivering information of mechanical environments surrounding cells to the nucleus transcriptional machinery. In this review, we discuss mechanical cue-dependent regulatory mechanisms for YAP/TAZ functions, as well as their clinical significance in cancer progression and treatment.

## Introduction

**Fig. 1:**
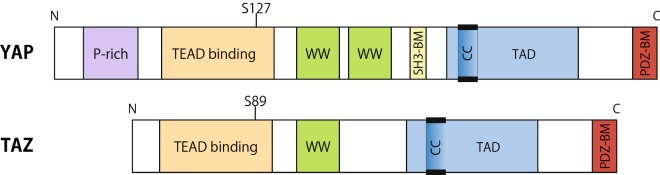
Domain structures of YAP and TAZ. YAP is a 65-kDa protein containing a proline-rich region (P-rich) in the N-terminal region and a PDZ-binding motif (PDZ-BM) in the C-terminal region that are separated by two WW domains, a TEAD binding domain, a Src homology domain 3 binding motif (SH3-BM), and a coiled–coil domain (CC) within the transactivation domain (TAD). There are 8 isoforms of YAP exist which differ by loss of one WW domain and alterations in TAD. TAZ, a 43-kDa paralog of YAP, has similar domain organization but lacks the proline-rich region, the SH3-BM and one WW domain. YAP S127 and TAZ S89 are the main phosphorylation targets of LATS1/2. Upon phosphorylation YAP/TAZ binds with 14-3-3 and thus are sequestered in the cytoplasm

The transcriptional coactivators YAP and TAZ (Fig. 1) have compelled researchers’ attention over the past decades as important mediators of multiple biological functions. YAP/TAZ play essential roles in development, homeostasis and regeneration of tissues and organs [1–6], and their dysfunctions cause several diseases [1, 4, 5, 7]. Both YAP and TAZ are expressed in most of human tissues with some exceptions, for instance TAZ is not expressed in thymus and peripheral blood leukocytes, while YAP is not expressed in hippocampus and parathyroid gland [8–10].

As a canonical mechanism for YAP/TAZ regulation, a phosphorylation cascade of the Hippo pathway has been identified [11]. There are two major steps in Hippo pathway-mediated regulation of YAP/TAZ: YAP/TAZ phosphorylation and nuclear translocation of YAP/TAZ. Nuclear YAP/TAZ mainly interact with transcription factors of the TEA domain (TEAD) family members, as well as several other transcriptional factors to regulate gene expression (reviewed in [12]; and [13–16]). While the Hippo pathway is the most widely known regulator of YAP/TAZ activity, it is not the sole one. Since the first identification of YAP/TAZ as a mechanotransducer [17], many groups have shown that YAP/TAZ are regulated in response to a number of different types of mechanical stimuli through Hippo-independent mechanisms.

In this review, we first discuss regulatory mechanisms of YAP/TAZ focusing on their regulation by mechanical cues from cell microenvironments such as adjacent cells and the extracellular matrix (ECM). We further discuss the roles of YAP/TAZ in tissue homeostasis and pathology with particular interest in their roles in cancer progression and resistance against therapeutic treatments.

Importantly, while YAP and TAZ have a lot of similarities in their structures, regulations and functions, they are not identical (Fig. 1; [8, 18, 19]). Therefore, in this review we use the term “YAP/TAZ” when there is evidence that YAP and TAZ share the described functions or regulations, whilst we specify the single protein name when evidence is available only for either YAP or TAZ.

## Canonical YAP/TAZ regulation via Hippo pathway

**Fig. 2:**
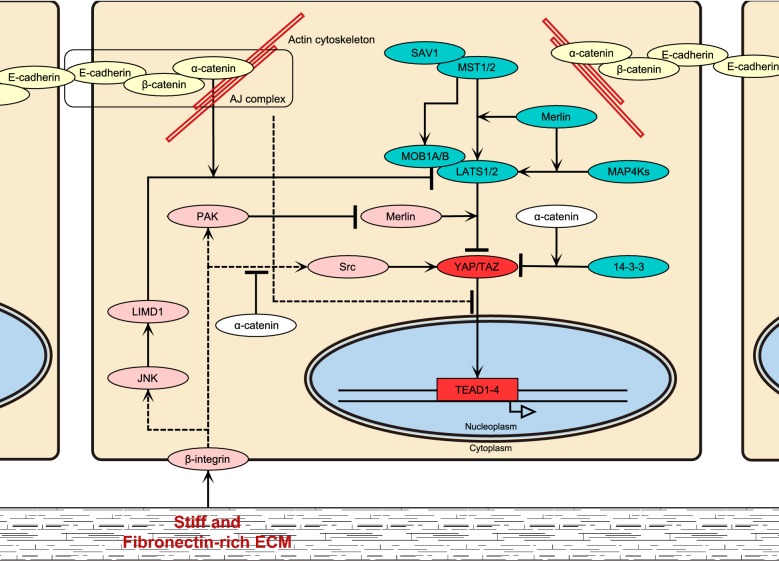
Overview of signaling cascades for YAP/TAZ regulation. Hippo pathway- (blue), FA- (pink) and AJ-mediated (yellow) regulations of YAP/TAZ are shown. See the main text for details

The core Hippo pathway starts with MST1/2—STE20 family protein kinases, which phosphorylate hydrophobic motifs of LATS1/2 [20]. Phosphorylated, i.e. activated, LATS1/2 then phosphorylate serine residues in YAP/TAZ [11, 21], leading to cytoplasmic retention of YAP/TAZ [21] (Fig. 2). In addition to direct phosphorylation of LATS1/2 by MST1/2, MTS1/2-phosphorylated SAV1 and MOB1A/B facilitate phosphorylation of LATS1/2 [22–24]. While the role of MOB1A/B is not fully understood (Meng et al. proposed a model in which MOB1A/B act as scaffold proteins, but it has not been experimentally justified yet [25]), SAV1 binds to MST1/2, targeting the whole complex to the plasma membrane [26]. For quite a long time, MST1/2 have been deemed to be the primary kinases that phosphorylate LATS1/2. However, it has been shown that double-depletion of MST1/2 does not alter YAP phosphorylation [27, 28]. Indeed, a recent study has shown that MAP4K family kinases act in parallel to MST1/2 to activate LATS1/2 [29].

Upon phosphorylation by LATS1/2, YAP/TAZ interact with 14-3-3, which sequesters YAP/TAZ from translocation into the nucleus [21]. α-Catenin, a well-known adaptor protein localizing at cadherin-mediated cell–cell junctions, stabilizes the YAP complex with 14-3-3, thereby inhibiting YAP dephosphorylation by protein phosphatase 2A (PP2A) [27, 30]. It has been suggested that α-catenin acts at adherens junctions (AJ) to stabilize the YAP complex [27]. However, in our opinion, α-catenin may interact with the YAP-14-3-3 complex both at and outside AJs. While this idea requires further justification, there are several observations supporting this idea. First, exclusion of YAP from the nucleus usually does not lead to its plasma membrane recruitment, rather it more or less evenly distributes throughout the cytoplasm [28, 31, 32]. Second, α-catenin does not always solely localize at AJs but shows significant cytoplasmic attendance [27, 33]. Last, α-catenin shows a protective effect against PP2A-mediated dephosphorylation of YAP in an in vitro dephosphorylation assay without membrane supports [27], indicating its ability to act as a soluble form. As such, the cytoplasmic complex formation with α-catenin would ensure retention of YAP in the cytoplasm.

Another mechanism for inhibiting nuclear localization of YAP/TAZ is phosphorylation of YAP/TAZ by casein K1δ/ε following the LATS-dependent phosphorylation. Such polyphosphorylation induces YAP/TAZ ubiquitination by the SCF E3 ubiquitin ligase and subsequent degradation [25].

To date, several upstream regulators of the Hippo pathway have been identified: TAO1/2/3 kinases that phosphorylate the activation loop of MST1/2 [25, 34, 35], G-protein coupled receptors [36], Kibra [37], PTPN14 [37], AMPK in response to energy stress [38–40], and Ras-mitogen activated protein kinase (MAPK) signalling [36, 41]. Moreover, while cells suffer various mechanical stimuli of different origins, including cell–ECM and cell–cell contacts, ECM stiffness, fluid shear, cell geometry, internal actomyosin tension and so on [1, 25, 42], these mechanical stimuli also strongly affect YAP/TAZ regulation.

## Impact of cell–ECM interaction on YAP/TAZ regulation

Cells adhere to ECM through the macromolecular adhesion complex called focal adhesion (FA) that links the actin cytoskeleton to ECM [43–45]. While cells sense stiffness of ECM and change their spreading and migration in response to ECM stiffness [17, 31, 46–52], YAP/TAZ activity is regulated by ECM stiffness and cell spreading. Spread cells and cells grown on stiff ECM show elevated YAP/TAZ activity which correlates with their nuclear localization [17, 31, 48, 53].

**Fig. 3:**
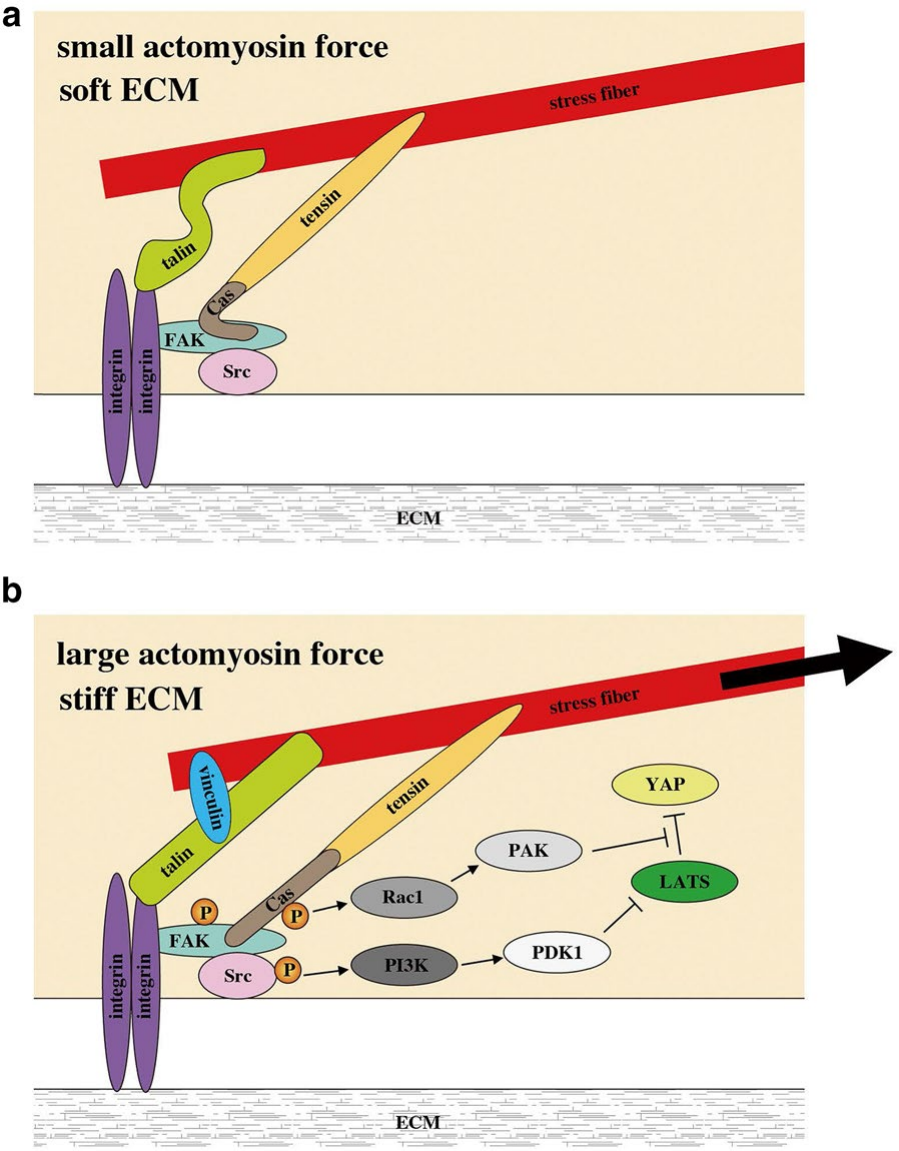
A hypothetical model for YAP/TAZ regulation by tension at FAs. **a** Low tension FA with small actomyosin force and/or soft ECM. **b** High tension FA with large actomyosin force and stiff ECM. FA tension induces sequential phosphorylation (P) of FAK, Src and p130Cas (Cas). Phosphorylated Src activates the PI3K–PDK2 pathway, whilst the phosphorylated p130Cas activates the Rac1–PAK pathway. Activation of these pathways inhibits LATS-mediated phosphorylation of YAP, facilitating nuclear translocation of YAP

Mechanical environments characterized by cell morphology and the cell–ECM contact area regulate YAP nuclear localization via affecting LATS1/2-dependent YAP phosphorylation [53]. As an underlying molecular mechanism, involvement of integrin signalling has been revealed (Figs. 2 and 3). Stiff or fibronectin-rich ECM activates the β1-integrin–FAK–Src–PI3K–PDK1 pathway, which inhibits the LATS1/2 activity and thus facilitates YAP nuclear translocation and transcriptional activation [54–56]. The Src–Rac1–PAK pathway also inhibits the YAP phosphorylation by LATS1/2 downstream of β1-integrin [57]. PAK activation induces phosphorylation of Merlin, which abrogates its scaffold function for YAP and LATS1/2, and thereby attenuates YAP phosphorylation by LATS1/2 [57] (Fig. 2). It is currently unknown whether FAK–Src–PI3K–PDK1 and Src–Rac1–PAK axes form a single cascade or act in parallel.

On the other hand, Dupont et al. reported that mechanical cue-induced regulation of YAP in human mammary epithelial cells and human mesenchymal stem cells is independent of the Hippo pathway [17], which was later confirmed by other groups [31, 48, 58]. However, for a long time, the mechanism that provides consistent explanation for the mechanical regulation of YAP/TAZ has been unsolved.

**Fig. 4:**
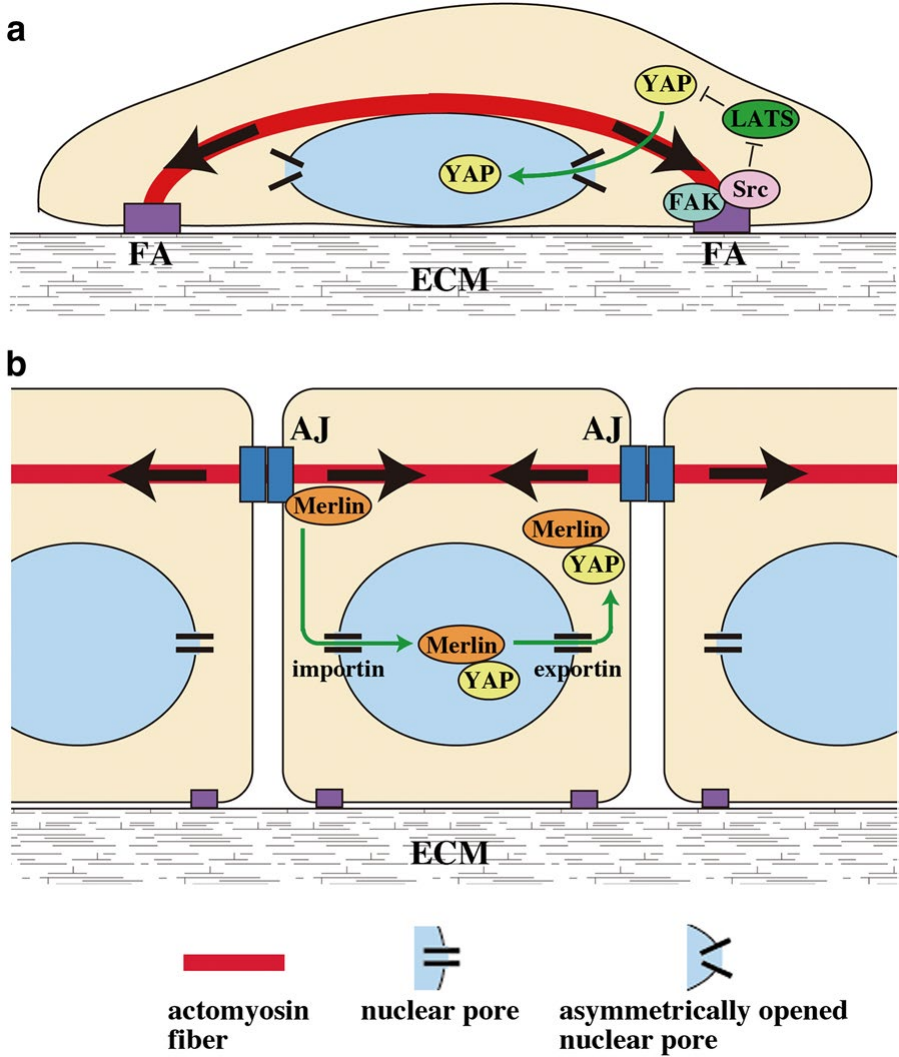
Hypothetical models for actomyosin-dependent regulation of YAP nuclear localization in sparse and confluent cells. **a** Sparse cells develop FAs that are connected to actomyosin stress fibers. Tensile force exertion from stress fibers to FAs activates the FAK–Src signal, which inhibits LATS1/2-mediated phosphorylation of YAP, thereby facilitating nuclear translocation of YAP. In addition, contraction of stress fibers connecting FAs and the apical surface of the nucleus (sometimes called ‘actin cap’) flattens the nucleus, increases the curvature of the lateral part of the nuclear membrane, and thereby enlarges the diameter of the cytoplasmic side of the nuclear pore in this nuclear membrane part. Such ‘asymmetric opening’ of nuclear pores may preferentially promote nuclear import, rather than export, of YAP. **b** Confluent cells are poor in FAs and stress fibers, but instead develop AJs and actomyosin fibers associated with AJs. Actomyosin-based tensile force at AJs induces translocation of Merlin from AJs to the nucleus, wherein Merlin forms a complex with YAP. With the aid of nuclear export signals of Merlin, the Merlin-YAP complex is then exported from the nucleus. Thus the actomyosin activity has opposing effects on the YAP distribution between sparse and confluent cells; the actomyosin activity promotes nuclear localization of YAP in sparse cells, but attenuates it in confluent cells. See detailed discussion in the main text

It took some time to find it, but recently Elosegui-Artola et al. have reported that the nucleus itself plays a role as a mechanotransducer for YAP regulation [59]. They have shown that the actin cytoskeleton, in particular actomyosin fibers running from FAs to the apical surface of the nucleus [60], affords mechanical connection between FAs and the Linker of the Nucleoskeleton and Cytoskeleton (LINC) complex of the nuclear envelope on stiff substrates, but not on soft ones. Contractile force generated by actomyosin activity flattens the nucleus and opens up nuclear pores. Such opening increases the rate of YAP/TAZ import but not affecting the export rate (Fig. 4a). This mechanism might also explain cell area- and cell shape-dependent regulation of YAP/TAZ localization [59].

As an attempt to explain the different influences of mechanical tension on import/export rates, the following model was proposed. The inner lumen of nuclear pores comprises a disorganized meshwork of flexible FG-nups that contain phenylalanine–glycine (FG) repeats [61, 62]. Repulsive interaction of proteins with FG repeats leads to protein unfolding and thus facilitates protein passage through the nuclear pore. Furthermore, tension in the cytoskeleton-nucleus system not only increases the nuclear pore size but also increases the curvature of the lateral part of the nuclear membrane. This leads to showing up the inner surface of the nuclear pore to the cytoplasm, thus promoting FG-nups-mediated translocation of cytoplasmic proteins into the nucleus (Fig. 4a). While their model could explain how nuclear import of YAP is facilitated, it does not fully explain why significant changes in the pore shape and size do not affect the export rate. Although we do not have a reasonable model that might explain all observations, it is necessary to take notice that the mechanism of nuclear transport is rather complex. In the case of YAP, its export from the nucleus would likely depend on the concentration of free nuclear YAP (i.e., YAP that is not trapped by TEAD [63] or other transcription factors in the nucleus), as well as availability of Merlin in the nucleus, wherein nuclear export signals of Merlin are suggested to mediate YAP nuclear export [64].

Nuclear localization of proteins is mediated by two distinct mechanisms: passive and active. Passive diffusion through nuclear pores is suitable for proteins smaller than ~ 40 to 50 kDa (note, theoretical MWs of YAP and TAZ are ~ 65 and ~ 43 kDa, respectively [18]). The second mechanism is the energy-dependent process that involves transporting proteins (importins and exportins). It is known that YAP/TAZ nuclear shuttling follows the active mechanism [17, 59, 64, 65]. Importantly, an increase in nuclear pore diameter under high tension in the actin cytoskeleton does not switch the mechanism of YAP/TAZ nuclear transport from active to passive; nucleus–cytoplasm translocation of YAP/TAZ under high tensile conditions (e.g., on stiff ECM) still depends on importin and exportin activities [59].

Thus at least two distinct mechanisms underlie cell–ECM interaction-mediated activation of YAP/TAZ: inhibition of LATS1/2-mediated phosphorylation of YAP/TAZ, and an increase in YAP/TAZ import through opening of the nuclear pores, which might synergistically regulate YAP/TAZ transcriptional activity (Figs. 3 and 4a).

## Cell–cell contact-mediated regulation of YAP/TAZ

Sensing neighboring cells is essential for tissue integrity. Cell–cell contact-induced arrest of cell growth, termed contact inhibition of proliferation (hereafter contact inhibition, CIP), underlies homeostatic control of cell density and organ size [30, 66]. Involvement of YAP/TAZ in cell contact inhibition and tissue growth control has been revealed over the decade [21, 30, 67]. However, the detailed mechanism underlying YAP/TAZ-mediated CIP is still poorly understood.

At a low cell density condition the phosphorylation level of YAP is low and it predominantly localizes in the nucleus, while at a dense condition YAP is highly phosphorylated and sequestered in the cytoplasm [17, 21, 64]. Under a high cell density condition, LATS1/2 are phosphorylated and activated by MST1/2 and MAP4K1/2/3/4/6/7 [17, 21, 28, 29], and double-depletion of LATS1/2 abrogates cell density-dependent phosphorylation of YAP [29], indicating that LATS1/2 are responsible for phosphorylation of YAP/TAZ in response to cell density.

Several studies have revealed important roles of AJ components in YAP regulation [27, 30, 32, 68, 69]. AJ is a macromolecular complex that mediates adhesion between cells and is composed of transmembrane cadherin family proteins (E-cadherin in the case of epithelia) and their associated catenins [70, 71]. The extracellular domain of cadherin is responsible for homophilic interactions of cadherin between neighboring cells. On the cytoplasmic side, β-catenin binds to the cytoplasmic domain of cadherin, as well as interacts with the actin binding protein α-catenin. It is well established that E-cadherin–β-catenin–α-catenin-actin linkage provides a major mechanical connection of the actin cytoskeleton to E-cadherin, which transmits actomyosin-generated tensile force to E-cadherin at AJs [72]. Stability and function of AJs depend on trans interaction of the cadherin extracellular domains and actomyosin-generated mechanical tension [71]. A number of other proteins have been shown to localize at AJs.

E-cadherin reportedly mediates CIP in a Hippo pathway-dependent manner [28]. While E-cadherin homophilic ligation in MCF-7 and MCF-10 cells leads to inhibition of cell proliferation, this process depends on other AJs components, including α-catenin, β-catenin, NHERF (Na^+^/H^+^ exchanger regulatory factor) and Merlin, as well as the Hippo pathway components Kibra and LATS1/2. Interestingly, MST1/2 are not required for this process.

Involvement of E-cadherin in the cell density-dependent regulation of YAP has been further supported by the findings that MDA-MB-231 cells, which do not express E-cadherin, show nuclear localization of YAP even under the high cell density condition, and that exogenous expression of E-cadherin in these cells redistributes YAP from the nucleus to the cytoplasm [28]. It is noteworthy that expression of the E-cadherin mutant, which cannot bind to the catenin complex but is still able to mediate cell–cell adhesions, does not cause relocalization of YAP into the cytoplasm. By contrast, expression of an E-cadherin/α-catenin chimeric protein that can directly bind to the actin cytoskeleton induces cytoplasmic sequestration of YAP, even more efficiently than that of wild-type E-cadherin. These results suggest that connection of E-cadherin to the actin cytoskeleton contributes to sequestering YAP in the cytoplasm at a dense condition. Consistently, depletion of either β-catenin or α-catenin induces nuclear localization of YAP in dense cell cultures, as well as in mouse skin [28, 30]. Anyway, there is no doubt that E-cadherin is essential for cell density-dependent subcellular localization of YAP in epithelial cells.

**Fig. 5:**
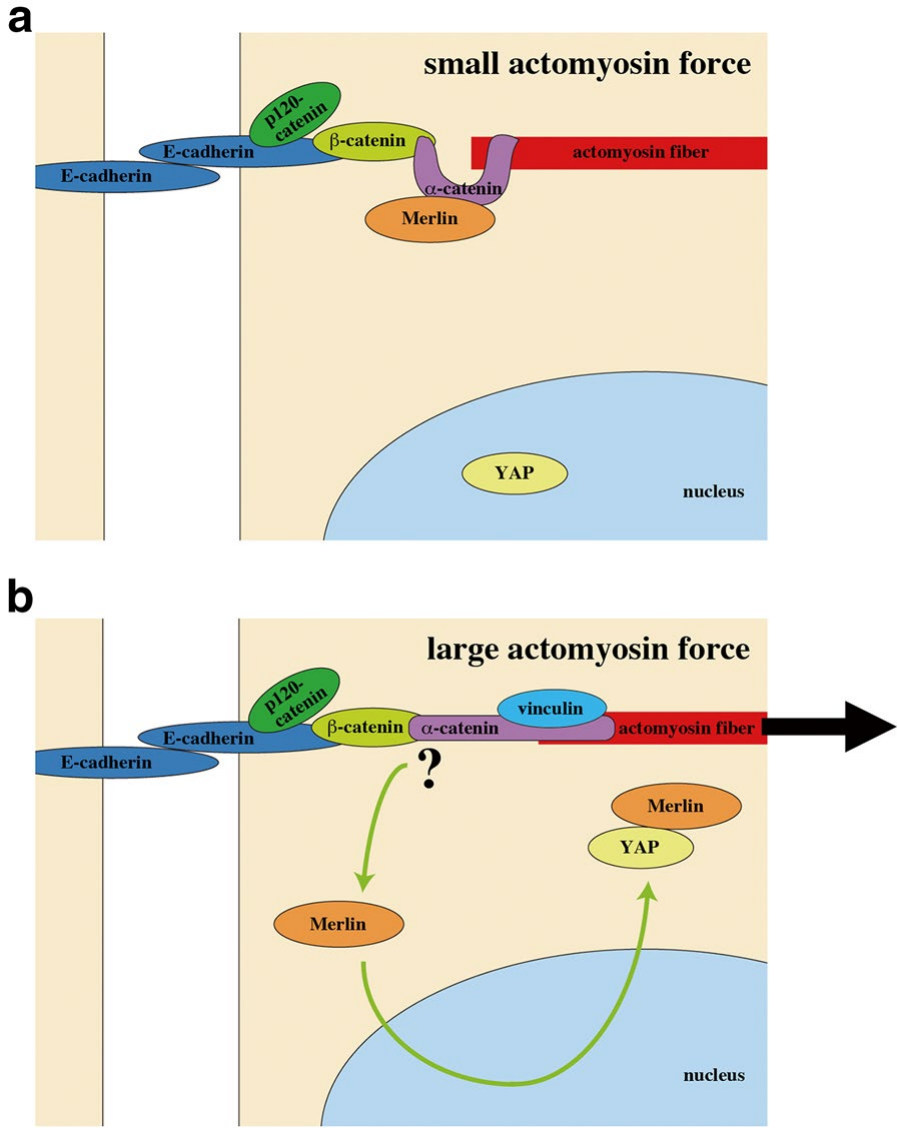
A hypothetical model for YAP/TAZ regulation by tension at AJs. **a** Low tension AJ with small actomyosin force. Under this condition, Merlin localizes to AJs through its binding with α-catenin. **b** High tension AJ with large actomyosin force. AJ tension causes dissociation of Merlin from AJs, which may be associated with a force-induced conformational change of α-catenin. Released Merlin enters the nucleus, binds nuclear YAP, and then exports it from the nucleus with the aid of NESs in Merlin

Recently, two independent groups have revealed that actomyosin-based tension at AJs suppresses nuclear localization of YAP/TAZ in high density epithelial cells, thereby arresting cell growth [32, 64] (Figs. 4b and 5). Thus inhibition of actomyosin or disconnection of the actin cytoskeleton to AJs (by depletion of α-catenin or β-catenin) causes nuclear localization of YAP in high density cells as well as in the mouse skin [28, 30, 32, 64]. Application of external tensile force to E-cadherin using E-cadherin-coated magnetic beads, in turn, arrests YAP-driven proliferation of actomyosin-inhibited cells, while E-cadherin homophilic ligation per se promotes cell proliferation under actomyosin inhibition [32]. Thus, considering importance of tension at AJs, we suggest that not E-cadherin by itself but mature AJs connected to actomyosin network would be required for cell density-dependent YAP regulation and CIP.

As a molecular mechanism for the AJ-tension-dependent regulation of YAP/TAZ localization, Furukawa et al. have shown that YAP/TAZ are excluded from the nucleus dependently on Merlin and its nuclear export signals (NESs) [64]. While Merlin associates with AJs, actomyosin-based tension at AJs releases Merlin from AJs and allows it to enter the nucleus. Nuclear Merlin binds YAP/TAZ and facilitates cytoplasmic translocation of YAP/TAZ with the aid of Merlin NESs (Fig. 4b). Of note, Merlin acts in a Hippo-independent manner; silencing of Merlin expression does not affect phosphorylation of LATS and YAP.

Detailed mechanism for tension-dependent release of Merlin from AJs remains to be revealed. However, it is known that Merlin localizes at AJs through the binding with α-catenin [73]. α-Catenin is currently the solely revealed mechanosensor at AJs that alters its molecular interaction through the force-induced conformational change [74, 75]. This leads to an intriguing possibility that tension-induced conformational changes of α-catenin may be involved in dissociation of Merlin from α-catenin at AJs, which needs to be tested in future studies (Fig. 5).

Even though Furukawa et al. have revealed that Merlin deactivates YAP/TAZ by the Hippo-independent mechanism, Merlin can regulate YAP/TAZ also through the Hippo pathway. Thus Merlin functions as a scaffold protein that binds to LATS1/2 and targets them to the plasma membrane in *Drosophila* and mammalian HEK293 cells [26]. Merlin-dependent LATS recruitment to the membrane facilitates LATS1/2 phosphorylation by MST1/2 and MAP4Ks, followed by YAP/TAZ phosphorylation by LATS1/2 [26, 29], which supports the Hippo-dependent regulation model. Hence, we could speculate that Merlin regulates YAP/TAZ by multiple ways and different mechanisms may prevail in different cells and under different conditions (Fig. 2).

Merlin-dependent regulation of intracellular localization of YAP/TAZ has been confirmed also in vivo. Conditional knockout of Merlin in mouse liver results in significant liver expansion which coincides with decreased phosphorylation and increased nuclear accumulation of YAP [76]. Both, impairment of Merlin-driven nuclear export of YAP/TAZ and increased YAP/TAZ nuclear import due to a decrease in YAP/TAZ phosphorylation would contribute to enhanced nuclear accumulation of YAP/TAZ under Merlin knockout. Notably, overgrowth of Merlin-deficient liver progenitor cells was shown to be independent of YAP [77]. Thus the overall mechanism of Merlin-dependent regulation of cell growth is still unclear, and further studies are needed to unveil it.

It is known that Merlin localizes to AJs through its binding to α-catenin, and silencing α-catenin expression leads to delocalization of Merlin from AJs [73]. Even though an increase in non-AJ Merlin would accelerate export of YAP from the nucleus [64], α-catenin depletion, instead, leads to an increase in nuclear YAP [32, 64]. This suggests that α-catenin regulates YAP localization not only through Merlin but also via other mechanism(s). As discussed in the former section (“Canonical YAP/TAZ regulation via Hippo pathway”), cytosolic α-catenin may prohibit nuclear translocation of YAP by forming a complex with YAP. Similarly, β-catenin also forms a cytosolic complex with YAP as the β-catenin destruction complex, which contributes to sequestering YAP in the cytoplasm [78]. These results suggest that AJ components act not only as AJ-associated forms but also as soluble forms in the regulation of YAP/TAZ localization.

As described above, tension at AJs leads to sequestration of YAP/TAZ from the nucleus in high density cells [32, 64]. On the other hand, AJ tension under a lower cell density condition, in which cells form AJs but still proliferate, may have opposing effects on YAP localization. Under such condition, tension at AJs activates vinculin to recruit the LIM protein TRIP6 to AJs, which sequesters LATS1/2 at AJs and thereby inhibits LATS1/2 activation by MST1/2 and MAP4Ks, resulting in a decrease in YAP phosphorylation [79]. Thus AJ tension potentially regulates YAP/TAZ both positively and negatively depending on circumstances, even though the mechanism by which it takes opposing tasks is currently unknown and needs to be revealed in future studies.

## Differential regulations of YAP/TAZ by cell–cell and cell–ECM interactions

The finding that actomyosin-based tension at AJs causes cytoplasmic sequestration of YAP/TAZ in high density epithelial cells may sound contradictory to previous studies showing that actomyosin contractility facilitates YAP/TAZ nuclear translocation in many types of cells [17, 31, 59]. However dominant types of the actomyosin cytoskeleton are different between confluent epithelial cells and the sub-confluent cells used in the previous studies; while sub-confluent cells show prominent stress fibers connecting to FAs, confluent epithelial cells are poor in stress fibers but develop actomyosin cables that associate with AJs [32]. We speculate that actomyosin-based tension might have opposite effects on YAP/TAZ localization depending on the cellular context. Thus tension at FAs induces FAK phosphorylation [80], which causes YAP/TAZ activation [54, 55], as well as “opens” nuclear pores for YAP/TAZ nuclear import [59] (Figs. 3 and 4a). On the other hand, actomyosin tension at AJs deactivates YAP/TAZ (Figs. 4b and 5). Even though actomyosin inhibition potentially eliminates both effects, it may cause YAP deactivation in sub-confluent cells that have developed FAs but not AJs. By contrast, actomyosin inhibition in confluent epithelial cells would mainly abrogate the AJ-dependent suppressive effect on YAP, leading to activation of YAP.

Detailed mechanisms of such differential regulation is currently unknown. However, based on several recent reports, we could suggest that differential roles of α-catenin in YAP/TAZ regulation might be involved. On one hand, phosphorylation of the Ajuba family protein LIMD1 by JNK induces the complex formation of LIMD1 with LATS1/2 [81–84], and switch of α-catenin to open conformation under actomyosin-generated tension at AJs leads to recruitment of LIMD1–LATS1/2 complex to AJs and thereby inhibits the kinase activity of LATS1/2, resulting in activation on YAP/TAZ [85]. On the other hand, while Src directly phosphorylates three tyrosine residues in the transcription activation domain of YAP, α-catenin antagonizes ECM-induced activation of the β4 integrin–Src pathway, leading to inactivation of YAP [86] (Fig. 2).

## YAP regulates mechanical properties of tissues

In the above sections, we have discussed how mechanical cues from ECM and actomyosin tension regulate YAP/TAZ transcriptional activity. Interestingly, reverse regulations also exist. It has been recently revealed that YAP increases actomyosin-based tension in tissues (mouse lung and fish embryo) by upregulating the RhoA-actomyosin axis [87, 88]. YAP promotes expression of the Rho guanine exchange factor (RhoGEF) ARHGEF17 that activates RhoA [87]. Expression of several regulators and components of the actomyosin cytoskeleton, including myosin IIB, myosin regulatory light chain 2 and filamin A, is also enhanced by YAP either transcriptionally or non-transcriptionally [87, 89, 90]. Given that actomyosin tension induces YAP activation in many types of cells (see previous sections), YAP and RhoA signals are likely to be mutually regulated via a feedback loop.

On the other hand, YAP reportedly upregulates expression of the Rho GTPase activating proteins (RhoGAP) ARHGAP18/29 which deactivates RhoA [88, 91]. Thus expression of both a RhoA activator (ARHGEF17) and RhoA inhibitors (ARHGAP18/29) is promoted by YAP. It is currently unclear how opposing RhoA regulations by YAP through different RhoA regulators are spatiotemporally coordinated to fine-tune actomyosin systems.

The mechanical property of ECM is also regulated by YAP/TAZ. In cancer tissues, activation of YAP/TAZ in cancer-associated fibroblasts (CAFs) promotes expression of the ECM proteins such as laminin and fibronectin, and induces stiffening of ECM, which contributes to maintenance of cancer stem cells and CAFs in cancer environments [89, 92–94]. This YAP/TAZ-mediated regulation of ECM would contribute to cancer progression and therapy resistance, as discussed below.

## YAP/TAZ in tissue homeostasis

The Hippo pathway was originally discovered in *Drosophila*. The effect of the YAP/TAZ on the organ size control is preserved among species from flies to mammals [5]. However, sensitivity to YAP/TAZ dysregulation varies among different organs/tissues. Tissue specific knockout of YAP in virgin mammary gland and intestine does not result in any disorders in tissue size and structure [95, 96], while double conditional skin-specific knockout of YAP and TAZ results in dramatic loss of hair [56]. TAZ deletion causes the polycystic kidney disease [97], whereas YAP deletion leads to hypoplastic kidneys [98] and a bile duct defect in liver, without general reduction in organ size [5]. It is noteworthy that in most studies, in vivo functions of YAP/TAZ have been examined by knocking-out either YAP or TAZ. Since their functions are largely overlapping each other, it is possible that the effect of TAZ knockout may be compensated by YAP and vice versa.

While knockout of YAP/TAZ does not give rise to apparent phenotypes in some organs in a steady state, they appear to play significant roles during growth phases of tissues. For example, hyperactivation of YAP leads to a failure in terminal differentiation of the mammary gland during pregnancy [95]. YAP activity is induced upon hepatectomy, which is most likely critical for liver regeneration [99, 100]. Furthermore, loss of YAP causes a reduction of the regenerative ability of intestine [96].

YAP/TAZ are highly active in progenitor and stem cells in multiple tissues and influence cell fate. Active nuclear YAP/TAZ promote proliferation and self-renewal of embryonic stem cells (ESC) [101–103] and basal keratinocytes [27, 48, 103], as well as osteocyte differentiation of mesenchymal stem cells (MSC) [104, 105]. On the other hand, cytoplasmic retention of YAP/TAZ results in neuronal differentiation of ESC [101, 102], adipocyte differentiation of MSC [104, 106], and terminal differentiation of keratinocytes [48, 56].

## YAP/TAZ in cancer

Dysregulation of YAP and TAZ could be observed in many tumors of different origins, including prostate cancer, cervical squamous cell carcinomas, meningioma, squamous cell carcinoma of skin, malignant mesothelioma, acute myeloid leukemia, and others (comprehensively reviewed in [107]). Curiously, cancer-associated, activating mutations of YAP/TAZ have not been found, and *YAP1* and *TAZ* genes are rarely amplified in cancer cells [108]. Instead, YAP/TAZ are often overexpressed and highly accumulated in the nuclei in cancer cells [107, 109]. This phenotype is preserved in many cancer tissues [110–113]. Apparently, YAP/TAZ overexpression promotes transcription of YAP/TAZ target genes [114, 115]. High YAP/TAZ activity drives proliferation, invasion and metastases of cancer cells [4, 116–119], and is often associated with poor prognosis [120, 121], therapy resistance, including resistance to chemotherapeutic drugs [118, 121–125], radiation [124, 126] and molecularly targeted therapies [127, 128], and relapse [107] of cancers. In hematological cancers, however, low *YAP1* expression has been shown to be predictive of a poor outcome of treatments [129].

YAP/TAZ activity is usually essential for tumor progression, even though activation of YAP/TAZ solely might not be sufficient for cancer initiation in some tissues [5, 95, 130–132]. Importantly, mutations in Hippo pathway components are rare in human tumors [133]. Mutations in Merlin and LATS1/2 may occur only in specific tumor types, such as mesothelioma, schwannomas, and meningiomas, but are not observed in most tumor types displaying elevated YAP/TAZ activity [107]. Based on these results, Zanconato et al. reasonably suggest and argue that Hippo signaling is not a sole and dominant mechanism for regulating YAP/TAZ activity in human tumors [107].

These considerations raise following questions: how YAP/TAZ are activated in cancer and what is the sequence of events downstream of YAP/TAZ activation? To date mechanisms for activation and action of YAP/TAZ in cancer are poorly understood. However, some cues have been revealed in recent studies.

As was mentioned above, YAP and β-catenin synergize in cell cycle progression. Importantly, YAP overexpression in combination with β-catenin in mouse liver leads to the development of hepatoblastoma [134], and YAP is required for cell survival in β-catenin-driven colon cancers [119]. In both cases, YAP forms a complex with β-catenin. Rosenbluh et al. have found that the β-catenin–YAP–TBX5 complex formation drives expression of antiapoptotic genes, including *BCL2L1* and *BIRC5* [119]. Consistent with this, in BRAF-mutated non-small cell lung cancer (NSCLC) and melanoma, YAP-dependent expression of *BCL2L1* allows cancer cells to escape from apoptosis under BRAF- or MEK-targeting therapy [127], even though it has not been examined whether β-catenin is involved in this process.

Another role of YAP/TAZ in therapy resistance of cancer was found in studies of BRAF V600E gain-of-function mutant melanomas. YAP/TAZ-dependent actin cytoskeleton remodeling and cell spreading promote an acquisition of a phenotype resistant against the treatment with the BRAF inhibitor PLX4032 (vemurafenib) in melanoma cells in vitro [128]. Even though cytoskeleton remodeling per se may not be sufficient for therapy resistance, it is possible, as discussed in the previous sections, that cell spreading would facilitate nuclear entry of YAP (and, most likely, other transcription factors and co-activators), and thus might promote cell cycle progression and inhibit apoptosis in cancer cells.

YAP plays a key role also in acquiring EGFR-tyrosine kinase inhibitor-resistant as well as cetuximab-resistant properties of cancers [135–139]. Although details of the underlying mechanism remain unknown, several transcriptional targets of YAP, including AXL [136, 140], ERBB3 [141], PD-L1 [139], are involved in acquisition of these chemo-resistant phenotypes of cancers.

Cell invasion and metastasis are typical properties of malignant cancers. Notably, these activities of cancer cells are also potentially promoted by YAP. Recently, Qiao et al. have proposed a mechanism for YAP-mediated cancer metastasis [91]. First, the high expression level of YAP in cancer cells promotes epithelial–mesenchymal transition (EMT) via reprogramming of gene expression to antagonize AJ assembly [90, 94], which leads to loss of cell–cell adhesions and apical-basal polarity, along with acquisition of mesenchymal motility. Importantly, overexpressing YAP in normal cells antagonizes maturation of AJs, but does not induce EMT [90], suggesting that YAP cooperates with some other cancer-associated factors to provoke EMT in cancer cells [94]. Next, YAP-driven actin cytoskeleton remodeling decreases the rigidity of cancer cells [91]. Soft cancer cells are easy to squeeze through ECM and extravasate through vasculature to achieve distal metastasis via the circulation [142]. Furthermore, YAP enhances the membrane-cytoskeletal integrity, increasing cell viability when cells travel through narrow spaces such as capillary vessels during metastasis [91, 143].

The question regarding the mechanism of YAP/TAZ activation in cancer might be even more complex, considering potential existence of cell–cell and cell–ECM crosstalks in cancer environments [144, 145]. Although melanoma cells in vitro undergo cell death upon the treatment with the BRAF inhibitor PLX4720, these cells are resistant to PLX4720 treatment in vivo [146]. In vivo treatment of melanoma with PLX4720 activates melanoma-associated fibroblasts (MAFs), leading to ECM remodeling [146]. Changes in ECM composition and stiffness cooperatively provide “safe havens” for melanoma cells, making them insensitive to PLX4720 treatment [146]. The effect of MAF-dependent ECM remodeling can be recapitulated in vitro. When co-cultured in collagen gel, MAFs isolated from PLX4720-resistant cancer tissues make melanoma cells resistant to PLX4720 through formation of dense collagen fibers [146]. Importantly, melanoma cells re-isolated from the co-culture system remains sensitive to PLX4720. Significance of the ECM property has been further demonstrated by the observation that melanoma cells become PLX4720-resistant when solely cultured on stiff and fibronectin-rich substrates in vitro [146].

**Fig. 6:**
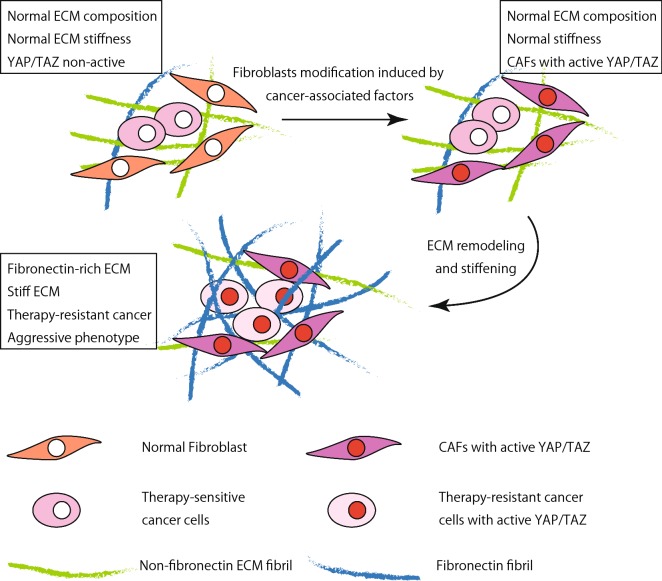
A hypothetical model of CAF-mediated YAP activation in cancer cells via ECM remodeling. On the first step soluble factors secreted by cancer cells activate YAP in CAFs, leading to ECM remodeling and stiffening. Stiff and fibronectin-rich ECM would promote YAP activation in both CAFs and cancer cells, which confers therapy resistance of cancer

Generation of the fibronectin-rich stiff matrix induces reorganization of integrin β1 into focal adhesions, elevating FAK phosphorylation [54, 146]. As discussed in the previous sections, it is conceivable that stiff ECM and phosphorylated FAK activate YAP/TAZ either via the Hippo pathway or independently of it. Similar ECM remodeling by mammary adenoma- and carcinoma-associated fibroblasts has been observed [89, 147], and ECM stiffening is a common feature of most solid tumor [148]. Thus, in a wide range of cancers, YAP might be regulated by the crosstalk between CAFs, ECM and cancer cells, as described in “YAP regulated mechanical properties of tissues”; YAP activity in CAFs might induce ECM remodeling that in turn would induce YAP nuclear translocation and phosphorylation by Src family kinases in cancer cells (Fig. 6). Although, the initial trigger that starts up this self-inducing program stays unknown, several studies support an idea that soluble factors secreted by cancer cells might act as such a trigger [36, 89, 147, 149].

Considering involvement of YAP and TAZ in cancer development, progression and therapy resistance, these molecules and their upstream regulators would serve as potent targets for cancer therapy [18, 109, 123]. Kinases are usually deemed to be the best targets for small molecule therapeutics. However, most kinases in the Hippo pathway are tumor suppressors, and restoring functions of tumor-suppressor kinases in cancers is a challenging task [109]. Alternatively, Src, which directly phosphorylates the YAP activation domain [86], may provide a potential target.

Another approach for YAP/TAZ-targeted cancer therapy is modulating the actin cytoskeleton. Recent findings that actomyosin contractility in epithelial sheets induces nuclear exclusion of YAP and thereby inhibits cell proliferation [32, 64] (see “Cell–cell contact-mediated regulation of YAP/TAZ” section) offer a potential strategy for suppression of YAP-driven cell proliferation by exogenous activation of actomyosin. However, it is important to notice that global induction of the actin cytoskeleton contractility would have harmful effects such as hypertension and asthma. Therefore, proper drug dosage and targeted drug delivery would be critical to implement cancer therapy that targets actomyosin activity.

Inhibition of YAP/TAZ interaction with its transcriptional partners is the third potential strategy. From screening a library of FDA-approved drugs targeting the YAP–TEAD complex, Liu-Chittenden et al. identified verteporfin (VP) as one of the top hits [150]. While VP is already used clinically for treatment of a non-cancer disease (macular degeneration), results in vitro and in mouse cancer models indicate VP as a promising component of synthetically lethal strategies in melanoma and NSCLC treatments in combination with vemurafenib and erlotinib, respectively [141, 151]. A peptide derived from vestigial-like protein 4 (VGLL4), a protein that competes with YAP in binding to TEAD and as such inhibits YAP function, also shows a therapeutic potential for cancers [152, 153]. In addition, flufenamic acid was recently identified as another inhibitor of YAP–TEAD-dependent transcription [154]. In contrast to the case of the YAP–TEAD complex, inhibitors of YAP interaction with other transcriptional factors are not known. However, as was mentioned above, YAP/TAZ-mediated transcriptional complexes independent of TEAD (such as the β-catenin–YAP–TBX5 transcriptional complex) might also drive the cancer progression and therapy resistance. Therefore, identification of inhibitors of TEAD-independent YAP/TAZ transcriptional complexes would contribute to development of novel therapeutic strategies for cancer.

Although pro-oncogenic functions of YAP/TAZ have been revealed by numerous researches, it is worth noting that there are several reports suggesting that YAP can function as a tumor suppressor and is required for p73-mediated and cisplatin-induced cell death [18, 155, 156]. For development of YAP/TAZ-targeted therapeutic strategies, further investigations are needed to reveal YAP/TAZ functions in different cancer types.

## Conclusion

Obviously, activity of YAP/TAZ is regulated by multiple mechanisms. It is also worthy to note that each cell suffers multiple mechanical and chemical stimuli from different origins. Therefore, multiple signaling cascades initiated by individual inputs may act in parallel, synergize to achieve stronger responses, or antagonize each other with prevailing of one of the mechanisms. Such pleiotropic regulations would fine-tune the YAP/TAZ transcriptional activity, enabling to control diverse biological functions of YAP/TAZ.

The important roles of YAP/TAZ in cell cycle progression, tissue growth and homeostasis make these proteins potential targets for clinical application, and one of the most straightforward application would be cancer treatment. Given that YAP/TAZ are regulated by multiple ways, better understanding of upstream regulators for YAP/TAZ is essential for developing therapeutic approaches that target YAP/TAZ signaling. Furthermore, as transcriptional co-activators, YAP/TAZ interact with various transcription factors to induce expression of a wide range of genes, some of which drive disease progression, and others of which might have protective effects against pathogenesis. Therefore, further studies aiming at exhaustive investigations on YAP/TAZ downstream effectors would be required for creating therapeutic strategies with high selectivities and efficacies.

## Abbreviations

AJ: adherens junction
CAF: cancer-associated fibroblast
CIP: contact inhibition of proliferation
ECM: extracellular matrix
EMT: epithelial–mesenchymal transition
ESC: embryonic stem cell
FA: focal adhesion
MAF: melanoma-associated fibroblasts
MSC: mesenchymal stem cell
MW: molecular weight
NES: nuclear export signal
NSCLC: non-small cell lung cancer
PP2A: protein phosphatase 2A
RhoGAP: Rho GTPase activating protein
RhoGEF: Rho guanine exchange factor
TAZ: WW domain containing transcription regulator 1
TEAD: TEA domain
VP: verteporfin
YAP: Yes-associated protein
